# Electrically Controlled Structures in Cholesteric Droplets with Planar Anchoring

**DOI:** 10.3390/molecules30224482

**Published:** 2025-11-20

**Authors:** Oxana O. Prishchepa, Mikhail N. Krakhalev, Anna P. Gardymova

**Affiliations:** 1 Kirensky Institute of Physics, Federal Research Center “Krasnoyarsk Science Center of the Siberian Branch of the Russian Academy of Sciences”, Krasnoyarsk 660036, Russia; kmn@iph.krasn.ru (M.N.K.); gard@iph.krasn.ru (A.P.G.); 2 Institute of Engineering Physics and Radio Electronics, Siberian Federal University, Krasnoyarsk 660041, Russia

**Keywords:** cholesteric liquid crystal droplet, planar anchoring, orientation structure, optical texture, electric field

## Abstract

Structure transformations in cholesteric droplets with planar anchoring induced by an electric field are studied experimentally. The radial spherical structure is formed initially, then it transforms into the quasi-nematic untwisting state under the action of an electric field E=1.75 V/μm. The dependence of structure transformations on the voltage switching-off mode is examined. At the one-step voltage-off mode, the Lyre structure is realized in cholesteric droplets at the relative chiral parameter in the range 4.3≤N≤8.5. The axis-symmetric bipolar structure and the low-symmetric planar bipolar structure are obtained at the multi-step voltage-off mode. The possibility of forming such structures and their stability are determined by the type of voltage switching-off mode, the *N* value, the surface anchoring strength (the value of cholesteric helix pitch), and the presence of the surface point defects.

## 1. Introduction

Cholesteric liquid crystals (CLCs) have a helicoidal ordering in their free state. CLC within a closed cavity can form a rich variety of orientational structures owing to interaction with a confining surface. According to the boundary conditions and the ratio of the cavity size to the helix pitch of CLC *p* (*p* is the distance at which the director turns by 2π), it is possible to form both the strong frustrated structures with a large number of defects [1], and the structure similar to an ideal helicoid one [2,3]. Hence, such CLCs are useful, for example, in biosensors [4,5,6], photonics [7], microlasers [8], reswitchable mirrors and smart films [9,10], unclonable markers [11,12], etc.

Polymer dispersed cholesteric liquid crystal (PDCLC) films are one of the kinds of soft matter that attracts interest from researchers up to now because of their producibility, usability, and a wide application [13,14,15,16]. Macroscopic optical properties of PDCLC depend mainly on the orientational structure of CLC inside droplet that can be operated with an electric field [17,18,19], mechanical affect [10,20], radiation [21], temperature variation [22]. The response mode of PDCLC is determined by the initial orientational structure, which is assigned by both the boundary conditions and the value of the relative chiral parameter N=2d/p, where *d* is the droplet diameter. At that, several various stable and meta-stable structures appear under identical conditions.

The quantity of possible structures is specified by the boundary conditions and the *N* value. As a rule, only one configuration is formed in CLC droplets when N≤2. These structures are the weakly twisted variant of the nematic ones: the twisted bipolar structure (BS) under tangential anchoring [23], the twisted radial structure under homeotropic anchoring [1], and the twisted axial-bipolar structure under conical anchoring [24]. Several layer-like structures, distinct by the topological defects in the droplet bulk and/or by the distortion manner of cholesteric layers, are realized in CLC droplets at N>10. Here, the thickness of the cholesteric layer is equal to the distance at which the director rotates by π, i.e., it is half of the helix pitch [2]. For example, the radial spherical structure (Frank-Pryce model) (RSS), the diametrical spherical structure (DSS), or the structure of nested cups [2,25] can be formed at the tangential anchoring. In the case of homeotropic boundary conditions, six layer-like structures can arise, distinguished by a number of points (poles) at the droplet surface where the normals to the cholesteric layers converge: the homogeneous twisted structure, the structure with one, two (bipolar distribution of helix axis), three, and four poles, and the fingerprint structure [26].

The richest variety of structures are observed at intermediate values 2<N<10. Under planar anchoring seven different configurations are formed: the twisted bipolar structure (BS), the bend-twisted bipolar structure, the planar bipolar structure (PBS), the Lyre structure (LS), the Yeti structure (YS), as well as RSS and DSS [27,28]. At homeotropic boundary conditions it is possible to form the structures with point defects in the bulk, the number of which varies from one to nine [1,29], the twisted toroidal configuration [19], the structure with bipolar distribution of the helix axis [17], and the structures with distorted linear defect and λ disclinations [30,31].

The structures’ diversity formed in the CLC droplets allows switching the whole PDCLC film between different states. To realize such an idea, it is necessary to find the switching manners of the droplets between the possible meta-stable states. For example, one can change the cholesteric helix pitch [32] or heat the CLC droplets to the isotropic phase and then cool rapidly to the CLC state [33], as well as create the temperature gradient [34]. In practice, the more convenient approach is the application of an electric field. In this case, selecting the modes of switching on/off the electric field, the various meta-stable structures can be obtained [35]. This approach was particularly applied to CLC droplets with homeotropic boundary conditions, and it enabled the switching between structures with the bipolar distribution of the helix axis and with the $\lambda^{+1/2}$ disclination in the bulk [31]. Moreover, new previously unobservable meta-stable structures were found, for example, the structure with the $\lambda^{-1/2}$ disclination [31], the structures with one or two cylindrical cholesteric layers, and the symmetric structures with several $\lambda^{+1/2}$ disclinations [35].

The response manner of CLC droplets with homeotropic anchoring depends, among other things, on the behavior of the linear defect present on the droplet surface. The point defects within CLC droplets with planar anchoring are formed at the surface, and consequently, one can expect other response dynamics and structure relaxation under switching on/off the electric field. At present, the response dynamics on applied voltage and relaxation of different structures, as well as electrically induced switching between meta-stable states, were not investigated in detail. In this work, we explore the structure transformations induced by switching the applied electrical field in CLC droplets with planar boundary conditions for the relative chiral parameter 4.0<N<13.0.

## 2. Results

### 2.1. Initial State

**Figure 1:**
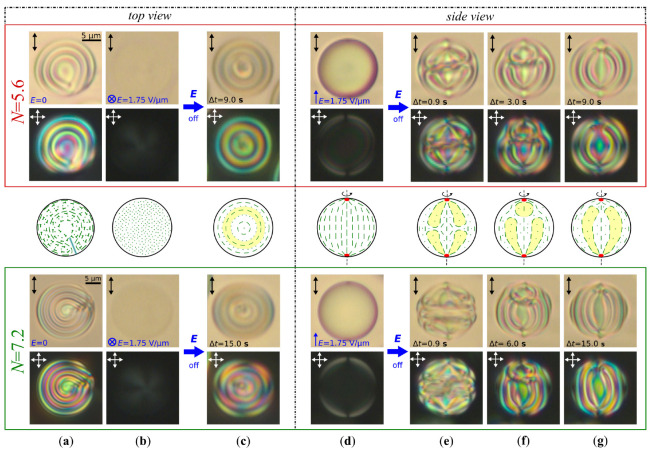
POM photos of CLC (p=5.6μm) droplets taken without analyzer (**top row**) and in the crossed polarizers (**bottom row**) for N=5.6 (in red frame) and N=7.2 (in green frame); (**a**) initial RSS (top view); (**b**) quasi-nematic state forming under E=1.75 V/μm applied perpendicular to the film plane (top view); (**c**) Lyre structures forming in Δt=9.0 s for N=5.6 and Δt=15.0 s for N=7.2 after switching off voltage; (**d**) quasi-nematic state forming under E=1.75 V/μm applied in the film plane (side view); (**e**) transient structures forming in Δt=0.9 s for N=5.6 and Δt=0.9 s for N=7.2 after switching off voltage (side view); (**f**) transient structures forming in Δt=3.0 s for N=5.6 and Δt=6.0 s for N=7.2 after switching off voltage (side view); (**g**) Lyre structures forming in Δt=9.0 s for N=5.6 and Δt=15.0 s for N=7.2 after switching off voltage (side view). Scale bars are 5μm. Schemes of director distribution corresponding to the CLC droplet at N=5.6 are presented between red and green frames. The red dots at the droplet’s poles in (**d**–**g**) are boojums. The areas confined by the closed cholesteric layers are marked by yellow.

The poly(isobutyl methacrylate) (PiBMA) polymer is a rather interesting material because it can assign various surface anchorings for different CLC mixtures. So, PiBMA sets homeotropic anchoring for the CLC based on E7 [19], while PiBMA specifies conical boundary conditions for the nematic LN-396 and the CLC based on it [24]. The PiBMA assigns the planar boundary conditions for CLC based on the nematic 6CHBT, as evidenced by the formation of RSS at N>4.0 (Figure 1a). The alternating lighter and darker isoclinic lines as concentric rings are revealed in the optical textures of the RSS droplets observed without an analyzer. The distance between adjacent lighter (darker) isoclinic lines is the cholesteric layer, equal to the half-pitch of the helix [2]. The distance between the isoclinic lines increases under the action of an electric field, and the structure gradually untwists (see Figure 5 in Ref. [36]). At high applied voltage, the CLC droplets transform into the quasi-nematic state characterized by the bipolar director orientation (Figure 1b,d). Such ordering has two surface point defects named as the boojums that are localized at the opposite droplet’s poles. The line joining the boojums (bipolar axis) is the symmetry axis of the structure [37].

In the following sections, the structure transformations of the electric-field-induced quasi-nematic state are examined for the one-step and multi-step voltage-off mode, as well as for three characteristic time periods: fast relaxation (Δt<1 s), slow relaxation (Δt∼10 s), and stabilization period (Δt∼10 h) [35]. Not all POM photos of observable structures are presented in both viewing directions (top and side). We selected only that view, where one can show the most distinctive features.

### 2.2. Structure Transformation at One-Step Voltage-Off Mode

Figure 1 shows the transformation of orientational structures in CLC droplets with N=5.6 and N=7.2 (p=5.6μm) resulting from the one-step electric field switching-off from E=1.75 V/μm to E=0 (see Movie S1 for N=5.6). Let’s consider the fast and slow relaxation stages of the structure in the CLC droplet with N=5.6. The boojums’ position is maintained for the fast relaxation period, and the CLC begins twisting from each boojum, so two closed cholesteric layers attached to the point defects are formed (Figure 1e). Then, during the slow relaxation stage, one closed layer expands at the cost of the second layer’s reduction in size (Figure 1f). Finally, the decreasing layer collapses, resulting in a formation of the closed cholesteric layer attached to one of the boojums and the second boojum at the opposite CLC droplet pole (Figure 1g). The closed cholesteric layer bends by 180° near the second boojum. This structure is symmetric relative to the bipolar axis, as evidenced the CLC droplet texture (Figure 1c), and it is similar to the simulated meta-stable Lyre structure [28]. In our experiment, the LS is meta-stable too, and its lifetime varies from several hours to several days at N=5.6 (p=5.6μm). Symmetry of the LS structure is broken during relaxation; the boojums are placed towards each other, resulting in the radial defect forming (see Supplementary Figure S1).

In the CLC droplet with N=7.2, two closed cholesteric layers attached to the boojums are formed at the fast relaxation stage when the electric field is switched off from E=1.75 V/μm to E=0. Simultaneously, the area with additional director distortions dividing the closed cholesteric layers appears in the droplet center (Figure 1e). In the subsequent phase of slow relaxation, one of the closed layers is expanded towards the opposing boojum. Concurrently, the second closed layer and the transient area between the closed cholesteric layers reduce in size. At the same time, the structure near the droplet equator undergoes additional twisting (Figure 1f). As a result, the Lyre structure with one closed and one cylindrical cholesteric layer is formed (Figure 1g). The position of the bipolar axis keeps during the fast and slow relaxation stages, and the lifetime of the LS in the CLC droplets with N=7.2 (p=5.6μm) is some minutes, then it transforms into the RSS.

**Figure 2:**
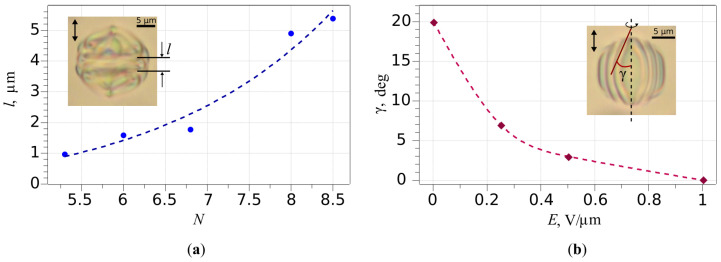
(**a**) Dependence of the thickness of intermediate layer *l* versus *N*; (**b**) Dependence of γ angle of curvature of barrel-shaped cholesteric layers versus applied electric field *E*.

The thickness of intermediate layer *l* formed by additional distortions of the director field between two closed cholesteric layers during the fast relaxation stage increases from 1.0 to 5.4 as *N* rises from 5.3 to 8.5 (Figure 2a).

As a result, at N>8.5, the appearing intermediate layer blocks the expansion of both closed cholesteric layers. It leads to the structure symmetry breaking, accompanied by the boojums displacement and the formation of the RSS during the slow relaxation stage (see Supplementary Figure S2). The closed cholesteric layers are not formed in the small CLC droplets at N<4.3 during the fast relaxation stage, so the Lyre structure does not appear. Therefore, it has been demonstrated experimentally that the Lyra structure can be formed only in the CLC droplets at 4.3≤N≤8.5 (p=5.6μm) in the studied system. These data agree with the predicted range of the LS existence simulated in [28].

### 2.3. Structure Transformation at Multi-Step Voltage-Off Mode

#### 2.3.1. Bipolar Structure

**Figure 3:**
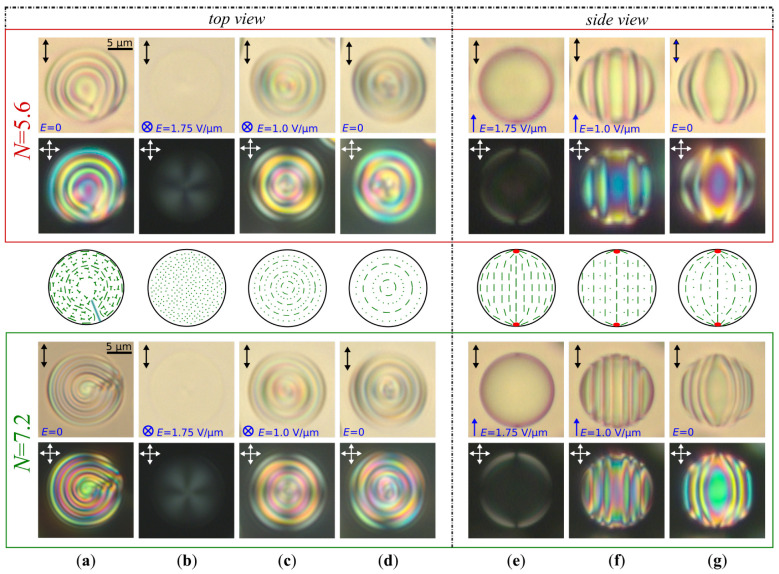
POM photos of CLC (p=5.6μm) droplets taken without analyzer (**top row**) and in the crossed polarizers (**bottom row**) with N=5.6 (in a red frame) and N=7.2 (in a green frame); (**a**) initial RSS (top view); (**b**) quasi-nematic state forming under E=1.75 V/μm applied perpendicular to the film plane (top view); (**c**) BS forming under the electric field reduction from E=1.75 V/μm to 1.0 V/μm (top view); (**d**) BS forming after switching-off electric field E=1.0 V/μm (top view); (**e**) quasi-nematic state forming under E=1.75 V/μm applied in the film plane (side view); (**f**) BS forming under the electric field reduction from E=1.75 V/μm to 1.0 V/μm (side view); (**g**) BS forming after switching-off E=1.0 V/μm electric field (side view). Scale bars are 5μm. Schemes of director distribution corresponding to the CLC droplet at N=5.6 are presented between red and green frames. The red dots at the droplet’s poles in (**e**–**g**) are boojums.

At the multi-step voltage-off mode, the electric field is initially reduced to a non-zero residual value lesser the structure untwisting value (the fast and slow relaxation stages), and then it is switched-off (Figure 3). The residual voltage value can be selected so that it will prevent the appearance of closed cholesteric layers attached to the boojums, as well as the intermediate layer formed by distortions of the director field. Consequently, the structure twists from the droplet equator, and the residual voltage unambiguously assigns an orientation to the cholesteric layers along the applied electric field (Figure 3b,c,e,f). Duration of residual pulse voltage should be more than the slow relaxation time. In our case, when the electric field is reduced from E=1.75 V/μm to E=1.0 V/μm, the cholesteric layers are formed as circular straight coaxial cylinders, the number of which is specified by *N* (Figure 3c,f). Such a structure is formed during approximately Δt≈1 s (see Movie S2). The cylinder axes coincide with the bipolar axis, which is oriented parallel to the residual electric field.

Switching off the residual voltage from E=1.0 V/μm to 0 results in a distortion of the cholesteric layers, which take barrel-shaped (Figure 3d,g) (see Movie S2 for N=7.2). Such a shape of cholesteric layers corresponds to the meta-stable BS [28]. If electric field E=1.0 V/μm is applied again to BS (Figure 3g), the cholesteric layers align to a cylindrical shape (Figure 3f). The curvature of barrel-shaped cholesteric layers can be operated by a value of applied voltage (Figure 2b). After switching off the electric field, the BS keeps for some hours. At the multi-step voltage-off mode, the BS is formed in CLC droplets with N<7.5 (p=5.6μm). After switching off the voltage, the axis-symmetric BS becomes unstable in the droplets with N>7.5. During a few seconds it transforms into a significantly deformed structure, relaxing into the RSS for a few minutes (see Supplementary Figure S3).

#### 2.3.2. Planar Bipolar Structure

**Figure 4:**
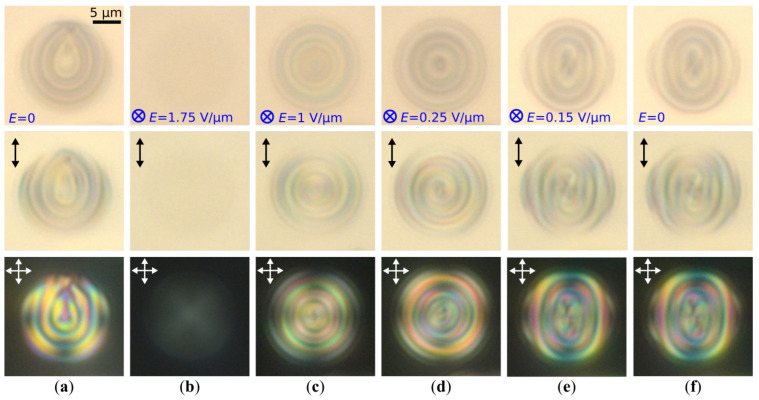
POM photos of CLC (p=5.6μm) droplets with N=6.0 taken in unpolarized light (**top row**), in polarized light without analyzer (**middle row**) and in the crossed polarizers (**bottom row**) (top view); (**a**) initial RSS; (**b**) quasi-nematic state forming under E=1.75 V/μm applied perpendicular to the film plane; (**c**) BS forming under the electric field reduction from E=1.75 V/μm to 1.0 V/μm, and (**d**) from E=1.0 V/μm to 0.25 V/μm; (**e**) PBS forming after the electric field reduction from E=0.25 V/μm to 0.15 V/μm, and (**f**) PBS after switching off the voltage. Scale bar is 5μm.

**Figure 5:**
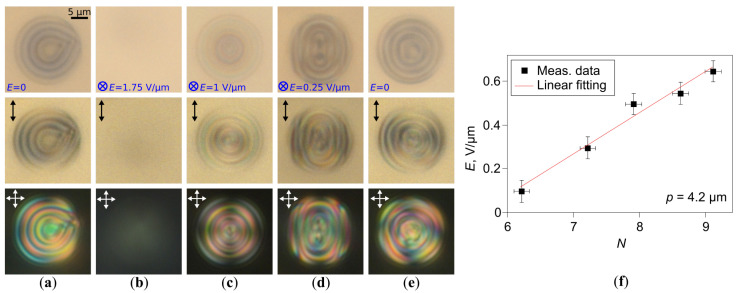
POM photos of CLC (p=5.6μm) droplets with N=7.3 taken in unpolarized light (**top row**), in polarized light without analyzer (**middle row**) and in the crossed polarizers (**bottom row**) (top view); (**a**) initial RSS; (**b**) quasi-nematic state forming under E=1.75 V/μm; (**c**) BS forming under the electric field reduction from E=1.75 V/μm to 1.0 V/μm; (**d**) PBS forming under the electric field reduction from E=0.35 V/μm to 0.25 V/μm; (**e**) BS forming after switching off the voltage. Scale bar is 5μm. (**f**) Dependence of electric field *E*, at which the BS transforms to the PBS on the *N* value (p=4.2μm).

As discussed above, the orientational structure in CLC droplets twists slightly at the abrupt switching-off of electric field from E=1 V/μm to 0, while the axial symmetry maintains (Figure 3c,d,f,g). In some cases, the twist of the CLC structure leads to a breaking of its symmetry. Such a transformation occurs as the residual electric field is gradually reduced. It results in the structure twist increasing from both sides of the droplet, and the shape of cholesteric layers becomes similar to the elliptical cylinder (Figure 4 and Figure 5). This process is accompanied by splitting the bipolar axis, and the BS transforms into the PBS. In the CLC droplets with N=6.0, the PBS is formed under E≅0.15 V/μm during approximately Δt≈10 s and persists for several hours after the voltage reduction to 0 (Figure 4e,f). The electric field strength at which the BS transforms into the PBS increases as *N* rises. For example, the PBS is formed in the CLC droplet with N=7.3 under E≅0.25 V/μm (Figure 5a–d).

After switching off the voltage, the PBS is observed within the cholesteric droplets at 6.0≤N≤7.5 (p=5.6μm), and its stability (lifetime) decreases as *N* increases. So, the PBS in CLC droplet with N≈6 preserves for days, and the PBS in the CLC droplet with N≈7 preserves for a few hours. Two distinct transformation scenarios of the PBS can be realized within the CLC droplets after switching off an electric field. In the first scenario, the structure symmetry becomes broken, and the PBS transforms into the RSS (see Supplementary Figure S4). In the second scenario, the PBS transforms inversely into the meta-stable BS (Figure 5d,e), which, in turn, can relax in the RSS.

The BS and PBS can be stabilized by the supporting voltage, which these structures keep during the action of the electric field. At that, an increase in the supporting voltage leads to a transformation of the PBS into the BS. Such a transition is observed at the same electric field strength at which the BS transforms into the PBS during the voltage reduction (see Supplementary Figure S5). The structure transformation accompanied by a formation of the BS and the PBS is also observed in the CLC droplets with the less helix pitch p=4.2μm. In this case, the BS and the PBS are more stable. The BS and PBS are formed and remain for several hours after switching off the supporting voltage in CLC droplets with the *N* parameter up to 13 (BS) and 10 (PBS). As shown experimentally, the dependence of electric field *E* at which the BS transforms to the PBS on the *N* is linear (Figure 5f).

A stability of the BS and the PBS is specified by both the *N* value and the CLC material parameters; therefore, a reduction in the helix pitch from p=5.6μm to p=4.2μm allows us to extend the range of *N* at which the BS and PBS are observed. It was numerically demonstrated that a decrease in the surface anchoring strength (droplet size at preservation of *N*) promotes the formation of the PBS [27]. In addition, a reduction in the anchoring energy by 20% is sufficient to make the PBS energetically more favorable compared to the asymmetric bend-twisted bipolar structure. In our case, decreasing the helix pitch by approximately 33% one can obtain the more stable PBS.

## 3. Discussion

The electric field-induced structure transformations in cholesteric droplets with planar anchoring have been studied experimentally. Initially, the radial spherical structure is formed in cholesteric droplets at N>4.0. Under the action of the electric field E=1.75 V/μm, the RSS untwists to the quasi-nematic state. Relaxation processes have been explored at both the one-step and multi-step voltage-off modes. At the one-step voltage-off mode, the meta-stable Lyre structure is obtained in the CLC droplets at 4.3≤N≤8.5. At the multi-step voltage-off mode, the axis-symmetric BS and low-symmetric PBS are realized. The stability of the BS and the PBS depends on the strength of supporting the electric field, the *N* values, as well as the CLC helix pitch. A low supporting voltage allows keeping BS/PBS during the action of an electric field. In the CLC droplets with helix pitch p=4.2μm, the BS and the PBS are more stable at the higher *N* compared to CLC droplets with p=5.6μm. It is caused by a stability dependence of these structures on the CLC material parameters and the surface anchoring strength. The proposed method to obtain the various CLC states is perspective to develop the switchable materials in which the orientation structure symmetry plays the key role in optical properties [38,39].

## 4. Materials and Methods

**Figure 6:**
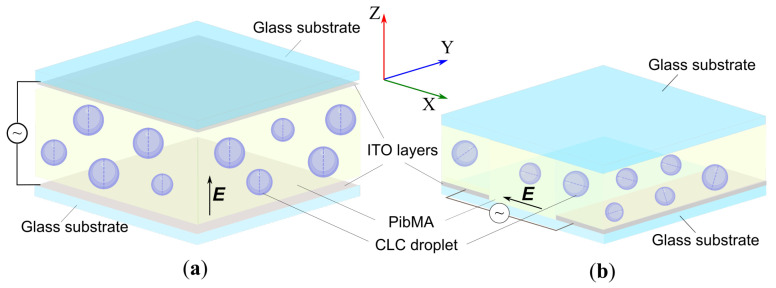
Schemes of experimental cells to explore the structure transformation in CLC droplets. (**a**) PDCLC film is sandwiched between two glass substrates coated with ITO layers, (**b**) PDCLC film is placed between two glass substrates. The bottom substrate is coated with an ITO layer divided by the non-conducting gap of approximately 100μm width, and the top substrate has no ITO layer.

The nematic liquid crystal 4-(trans-4’-n-hexylcyclohexyl)isothiocyanato-benzene (6CHBT) (Merck Group, Darmstadt, Germany) doped with the cholesteryl acetate (Sigma-Aldrich, St. Louis, MO, USA) and dispersed in poly(isobutyl methacrylate) (PiBMA) (Sigma-Aldrich, St. Louis, MO, USA) was studied. PDCLC films were manufactured by the method combining SIPS (solvent-induced phase separation) and TIPS (thermal-induced phase separation) [19]. CLCs with an intrinsic pitch of p=5.6μm (N<9.0) and p=4.2μm (N>5.0) were investigated. The CLC droplets were analyzed using the Axio Imager.A1m (Carl Zeiss AG, Göttingen, Germany) polarizing optical microscope (POM) at room temperature. The electrically induced modification of the CLC structures was examined in two types of cell: PDCLC film was sandwiched between two glass substrates coated with ITO (Figure 6a) and PDCLC film was sandwiched between the glass substrate coated with two ITO layers divided by a non-conducting 100μm gap and the glass substrate without ITO (Figure 6b). Consequently, an AC electric field of 1 kHz frequency and variable amplitude was applied perpendicular (for top view, along the *Z* axis) or parallel (for side view, in the YX plane) to the PDCLC film plane by the signal generator AFG-72105 (GW Instek, Taipei, Taiwan) combined with the amplitude amplifier AVA-1810 (Aktakom, Taipei, Taiwan). The thickness of the PDCLC film in both CLC cells was set by 20 μm diameter glass microspheres.

## Data Availability

The data presented in this study are available on request from the corresponding author.

## Supplementary Materials

The following supporting information can be downloaded at https://www.mdpi.com/article/10.3390/molecules30224482/s1: The structure transformation in the CLC droplet with N=5.6 (p=5.6μm) at the one-step voltage-off mode (Movie S1); the structure transformation in the CLC droplet with N=7.2 (p=5.6μm) at the multi-step voltage-off mode (Movie S2); and POM photos of the structure transformation of the LS to the RSS (Figure S1), the quasi-nematic state to the RSS (Figure S2), the BS to the RSS (Figure S3), the PBS to the RSS (Figure S4), and the BS to the PBS and vice versa (Figure S5).
