# Supplementary material for: Electrically Controlled Structures in Cholesteric Droplets with Planar Anchoring
Source: Molecules. 2025 Nov 20;30(22):4482. doi: 10.3390/molecules30224482 (PMC12655323; doi:10.3390/molecules30224482)
Supplement: Supplementary file 1 [file molecules-30-04482-s001.zip › Supplementary_Revised_Prishchepa.pdf]

Electrically controlled structures in cholesteric droplets with planar anchoring

Oxana O. Prishchepa[a,*], Mikhail N. Krakhalev[a,b], Anna P. Gardymova[a,b]

[a]*Kirensky Institute of Physics, Federal Research Center KSC SB RAS, 50/38 Akademgorodok, Krasnoyarsk, 660036, Krasnoyarsk region, Russia*

[b]*Institute of Engineering Physics and Radio Electronics, Siberian Federal University, 79 Svobodny Pr., Krasnoyarsk, 660041, Krasnoyarsk region, Russia*

∗Corresponding author.

Email address: p_oksana@iph.krasn.ru (Oxana O. Prishchepa)

Movie S1 –the structure transformation in CLC droplet with N = 5.6 (p = 5.6 μm) at the one-step voltage-off mode

Movie S2 – the structure transformation in CLC droplet with N = 7.2 (p = 5.6 μm) at the multi-step voltage-off mode

Figure S1-S5

Figure S1 shows a transformation of the meta-stable Lyra structure (LS) to the radial spherical structure (RSS) for hours (Figure S1).

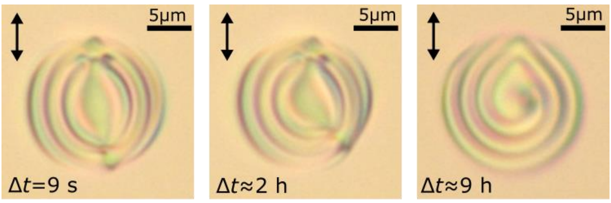

(a) ***(b)*** ***(c)***

Figure S1: POM photos of CLC droplet with Lyra structure (N = 6, p = 5.6 μm) taken in polarized light without analyzer in Δt = 9 s (***a***), in Δt ≈ 2 h (***b***), and in Δt ≈ 9 h (***b***) after switching off electric field.

Figures S2 demonstrates the relaxation process of CLC droplet (N = 8.57, p = 5.6 μm) from quasi-nematic state (Figure S2 a) to RSS (Figure S2 f) at one-step voltage-off mode. The structure with two cholesteric layers attached to the boojums, and the intermediate layer (Figure S2 b) is formed at abrupt switching off voltage. The structure symmetry is broken, accompanying by the boojums displacement and a formation of the transient structures (Figure S2 c-e). RSS is formed during the slow relaxation stage in Δt = 20.1 s after switching off voltage.

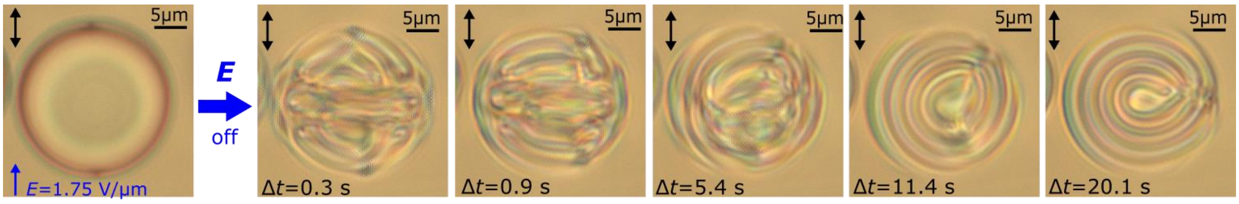

(a) ***(b)*** ***(c)*** ***(d)*** ***(e)*** ***(f)***

Figure S2: POM photos of CLC droplet (N = 8.57, p = 5.6 μm) taken in polarized light without analyzer; (***a***) quasi-nematic state forming under E = 1.75 V/μm applied in the film plane, (***b***) the transient structure forming in Δt = 0.3 s after switching off voltage; (***c***) the transient structure forming in Δt = 0.9 s after switching off voltage, (***d***) the transient structure forming in Δt = 5.4 s after switching off voltage, (***e***) the transient structure forming in Δt = 11.4 s after switching off voltage, (***f***) the RSS forming in Δt = 20.1 s after switching off voltage.

Figure S3 shows a transformation of unstable BS to stable RSS in CLC droplet (N = 8.8, p = 5.6 μm) during 20 s after switching off voltage E = 1.0 V/μm.

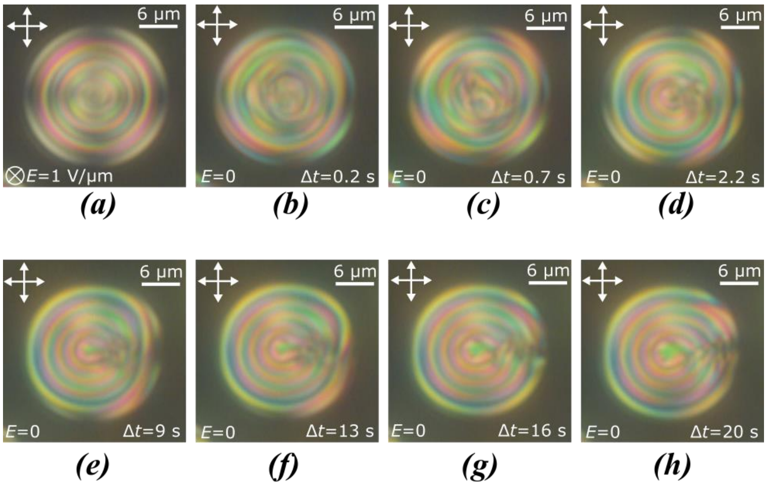

Figure S3: POM photos of CLC droplet (N = 8.8, p = 5.6 μm) under action of electric field E = 1.0 V/μm (***a***), and taken in 0.2 s (***b***), 0.7 s (***c***), 2.2 s (***d***), 9 s (***e***), 13 s (***f***), 16 s (***g***), and 20 s (***h***) after switching off voltage.

Figure S4 demonstrates a transformation of unstable PBS to stable RSS in CLC droplet ($N = 8.9$, $p = 5.6$ μm) during 30 min after switching off voltage $E = 0.50$ V/μm.

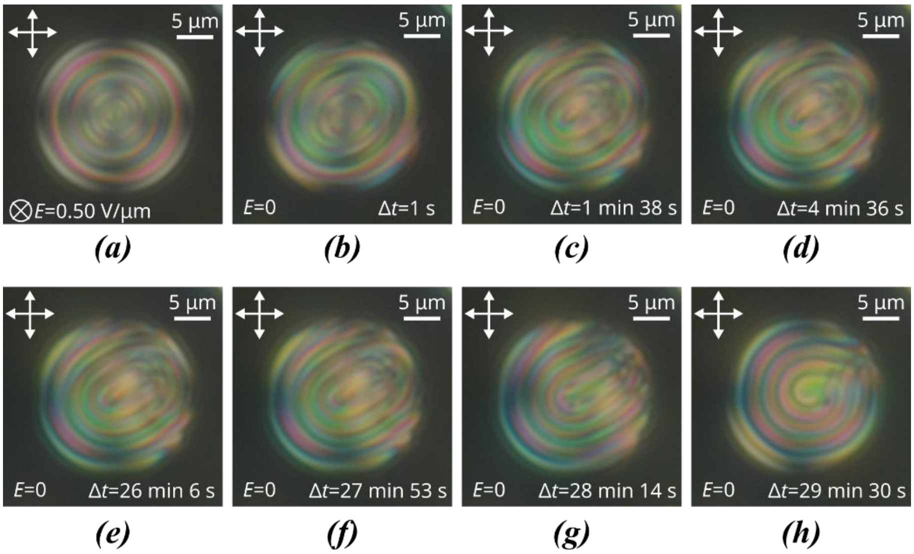

Figure S4: POM photos of CLC droplet ($N = 8.9$, $p = 5.6$ μm) under action of electric field $E = 0.50$ V/μm (***a***), and taken in $\Delta t = 1$ s (***b***), 1 min 38 s (***c***), 4 min 36 s (***d***), 26 min 6 s (***e***), 27 min 53 s (***f***), 28 min 14 s (***g***) and 29 min 30 s (***h***) after switching off voltage.

Figure S5 demonstrates the structure transformation in CLC droplet ($N = 7.4$, $p = 4.2$ μm). The initial RSS (Figure S5, a) transforms into the quasi-nematic state (Figure S5, b), then into the meta-stable structures at multi-step switching voltage (Figure S5, c-g). The BS is formed (Figure S5, c), the BS transforms into the PBS (Figure S5, d). The PBS keeps during hours after switching-off voltage (Figure S5, e). Increasing voltage up to $E = 0.3$ V/μm results in the PBS as before (Figure S5, f), and then the PBS transforms into the BS at increasing voltage to $E = 1.0$ V/μm.

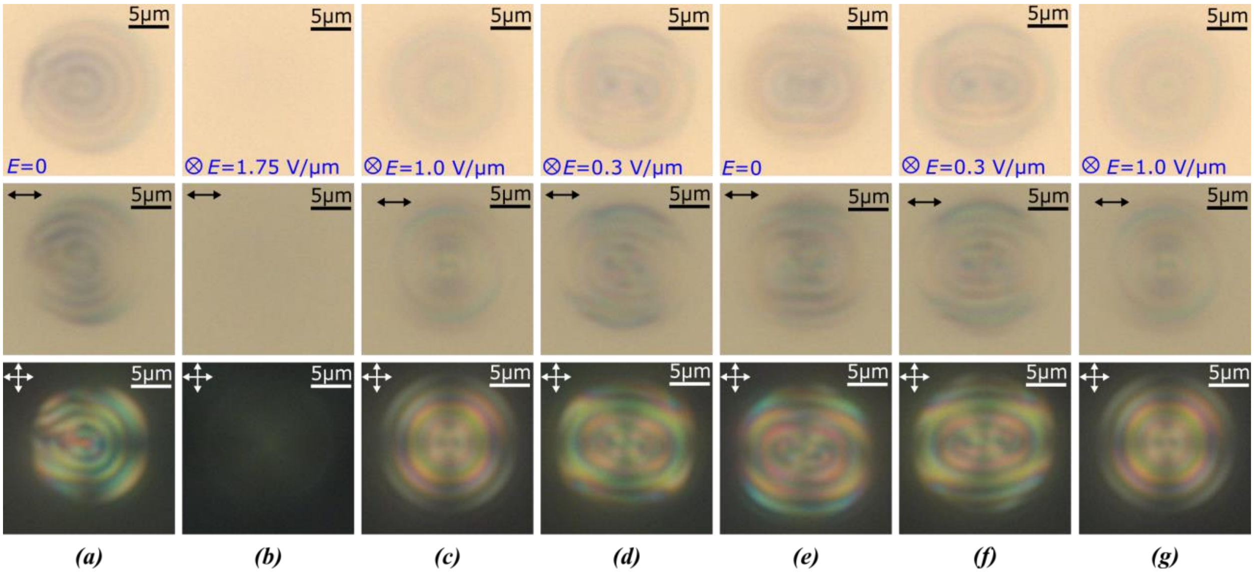

Figure S5: POM photos of CLC ($p = 4.2$ μm) droplets taken in unpolarized light (top row), in polarized light without analyzer (middle row) and in the crossed polarizers (bottom row) with $N = 7.4$; (a) initial RSS without voltage, (b) quasi-nematic state forming under $E = 1.75$ V/μm applied perpendicular to the film plane; (c) BS forming under $E = 1.0$ V/μm, (d) the meta-stable PBS forming after reduction voltage to $E = 0.3$ V/μm, (e) meta-stable PBS after switching-off voltage, (f) meta-stable PBS at increasing voltage to E = 0.3 V/μm, (g) BS forming at increasing voltage to $E = 1.0$ V/μm.
