# Supplementary figures and images for: Electrically Controlled Structures in Cholesteric Droplets with Planar Anchoring
Source: Molecules. 2025 Nov 20;30(22):4482. doi: 10.3390/molecules30224482 (PMC12655323; doi:10.3390/molecules30224482)

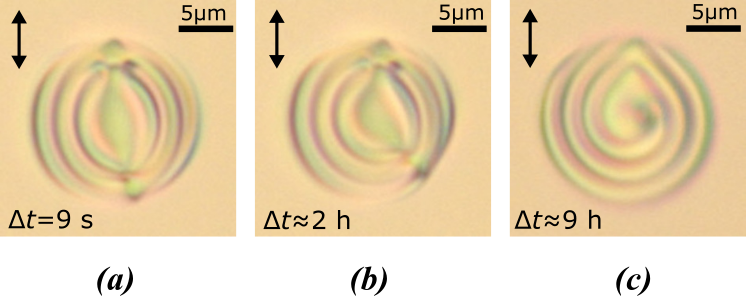

Supplement: Supplementary file 1 [file molecules-30-04482-s001.zip › Figure S1.png]

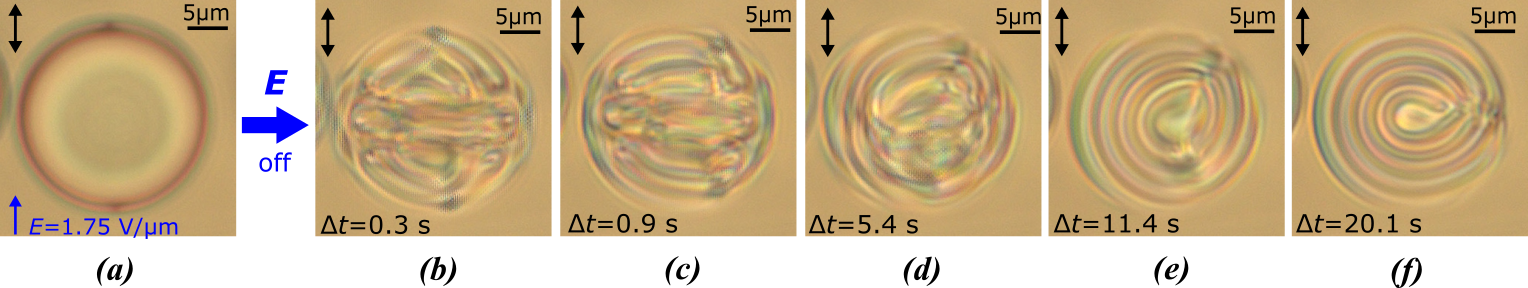

Supplement: Supplementary file 1 [file molecules-30-04482-s001.zip › Figure S2.png]

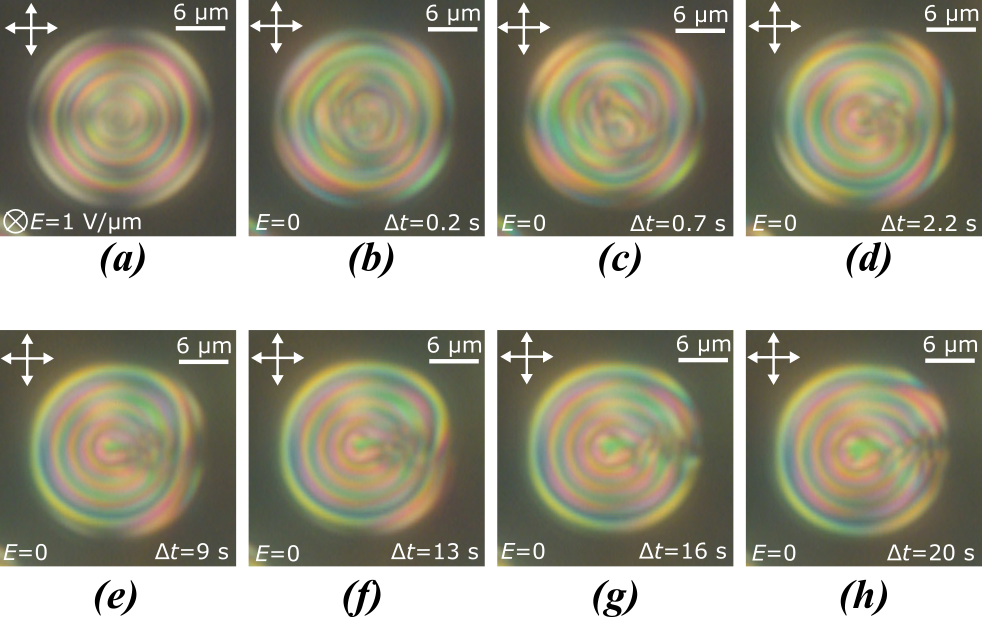

Supplement: Supplementary file 1 [file molecules-30-04482-s001.zip › Figure S3.png]

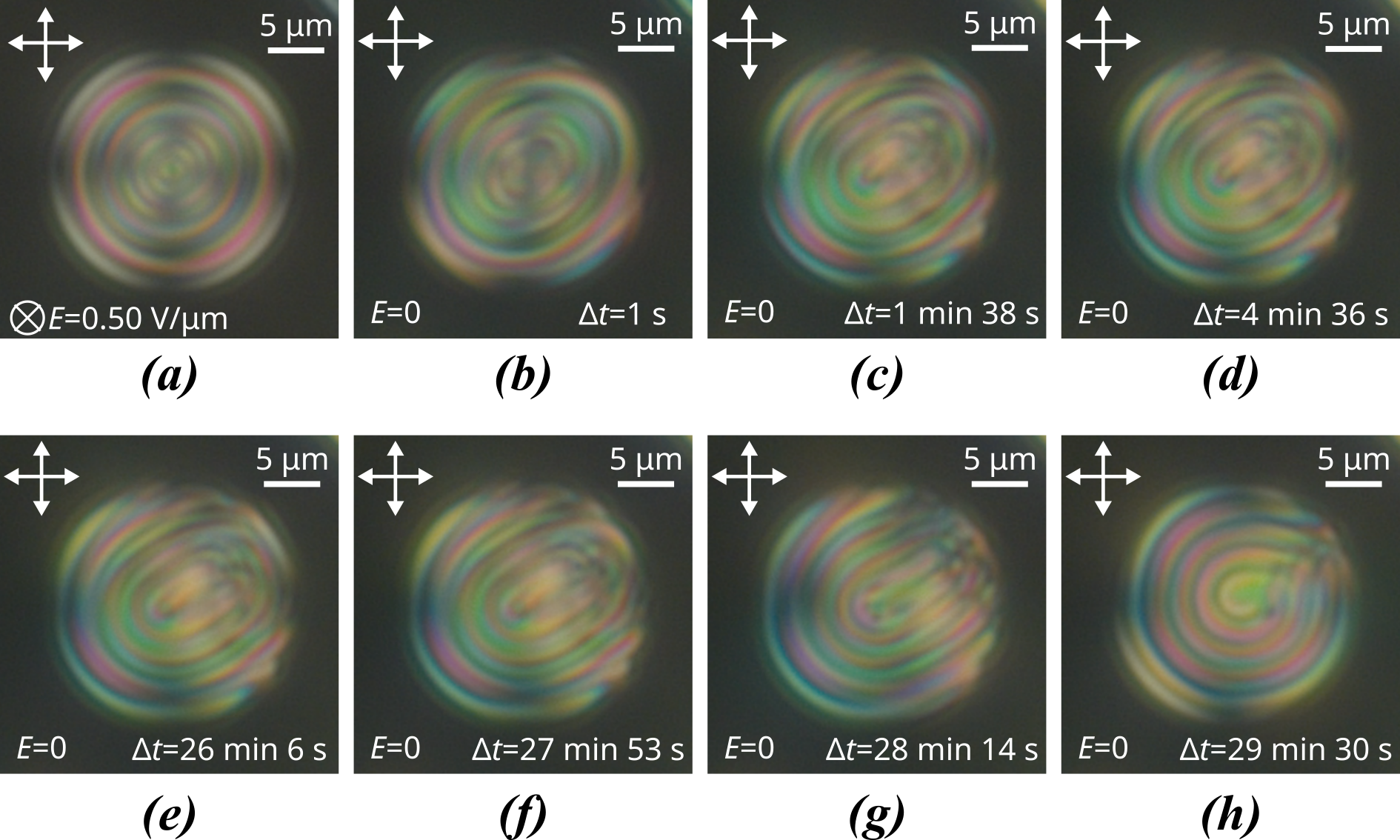

Supplement: Supplementary file 1 [file molecules-30-04482-s001.zip › Figure S4.png]

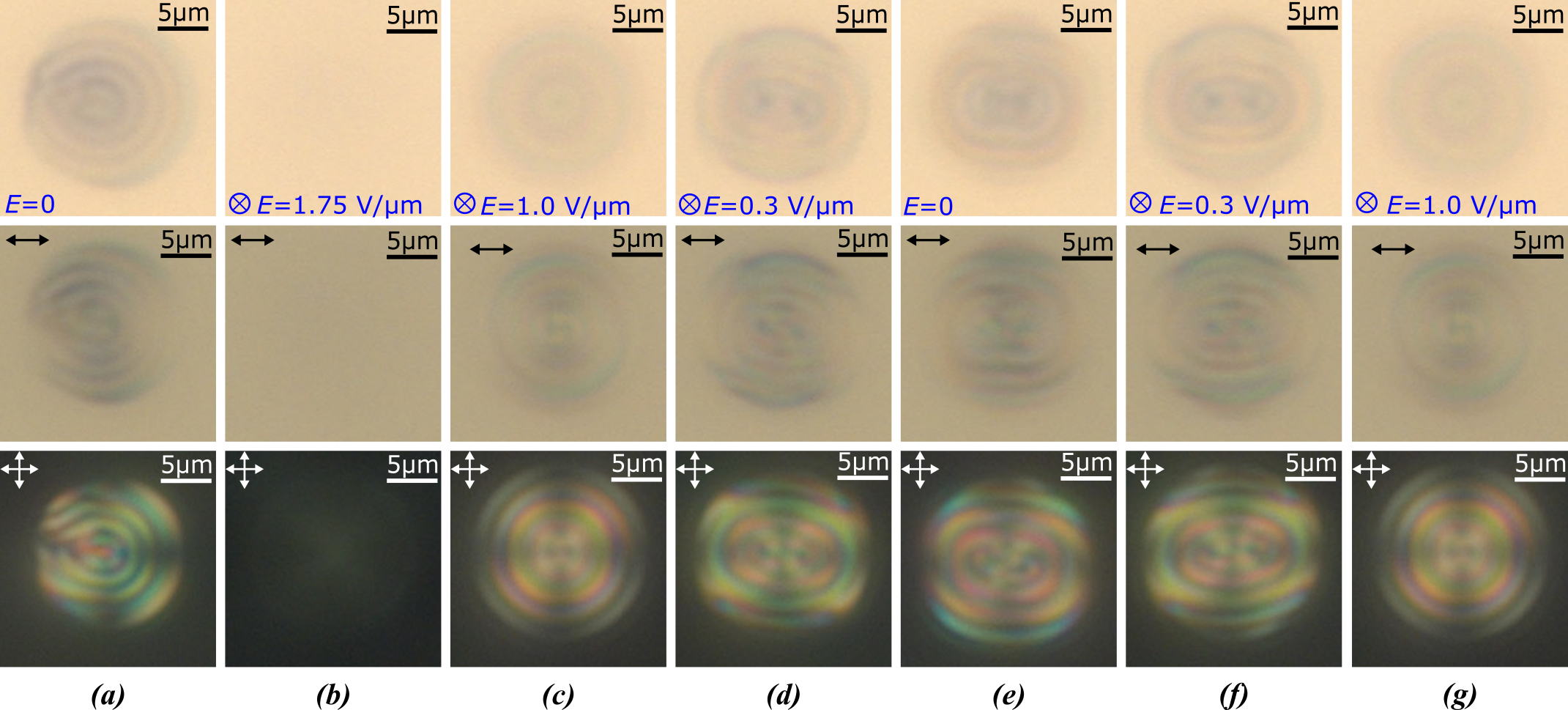

Supplement: Supplementary file 1 [file molecules-30-04482-s001.zip › Figure S5.png]
